# Longevity of Outcomes Following Reduction Mammoplasty

**Published:** 2019-07-23

**Authors:** Jennifer Bai, Carly M. Rosen, Ledibabari M. Ngaage, Colton H. L. McNichols, Silviu C. Diaconu, Chinezimuzo Ihenatu, Adekunle Elegbede, Emily C. Bellavance, Sheri S. Slezak, Yvonne M. Rasko

**Affiliations:** ^a^University of Maryland Medical Center, Baltimore; ^b^The Johns Hopkins Hospital, Baltimore, Md; ^c^The George Washington University Hospital, Washington, DC

**Keywords:** breast reduction, reduction mammoplasty, patient satisfaction, long-term outcomes, symptom relief

## Abstract

**Background:** Reduction mammoplasty has been shown to provide wide-ranging benefits for patients including improved quality of life in terms of physical function and mental health. However, most existing studies have been limited to the 1-year postoperative period. The aim of this study was to investigate long-term outcomes after reduction mammoplasty. **Methods:** Patients who underwent reduction mammoplasty at a single institution were identified retrospectively and grouped into 3 categories based on time since surgery: (i) 5 to 10 years, (ii) 10 to 15 years, and (iii) more than 15 years. A telephone survey was administered to measure satisfaction and symptom relief following reduction mammoplasty. **Results:** A total of 124 patients completed the survey and were included in the study. The majority of patients in all 3 groups reported marked symptoms relief (75% vs 82% vs 82%, *P* = .84). Overall satisfaction after reduction mammoplasty was high in all 3 subgroups and did not significantly decrease over time (4.16 vs 3.97 vs 3.7, *P* = .216) despite high proportions of patients reporting an increase in breast size since surgery (40% vs 70% vs 51%, *P* = .0297). **Conclusions:** Overall, reduction mammoplasty has long-lasting benefits for patients with macromastia. Overwhelmingly, patients report satisfaction with the procedure and marked symptom relief that is sustained for as long as 15 years after surgery.

Reduction mammoplasty is a common procedure performed to alleviate symptoms of macromastia. Symptoms resolved by reduction mammoplasty include back pain, bra strap shoulder grooving, intertrigo, posture problems, and breathing difficulty.^1^^-^3 In addition to relief of physical symptoms, studies have shown that reduction mammoplasty improves quality of life and provides psychosocial benefits regardless of preoperative risk factors.^4^^-^9

Patient perception of the results of the surgery and their overall experience are important considerations when working to improve patient care and surgical outcomes. To assess the benefits of reduction mammoplasty, patient-reported outcome measures have been increasingly used. For example, a retrospective analysis by Gonzalez et al^10^ utilizing the BREAST-Q concluded that reduction mammoplasty improves quality of life irrespective of preoperative body mass index (BMI). However, most existing studies have been limited to the 1-year postoperative period.

Thus, there is little evidence for long-term patient satisfaction following reduction mammoplasty and the longevity of quality of life effects due to mammoplasty has been questioned.^11^^-^15 Our study aimed to provide a better understanding of patient satisfaction and symptom relief from reduction mammoplasty over long periods of time.

## METHODS

A single-site retrospective review was performed to identify patients who underwent reduction mammoplasty at the University of Maryland Medical Center since 1999. Potential participants were identified using the *CPT* code 19318 for reduction mammoplasty and were included if they were 5 or more years out from surgery. Patients were divided into 3 groups based on years since surgery: (i) 5 to 10 years, (ii) 10 to 15 years, and (iii) more than 15 years since reduction mammoplasty at the time of survey. We excluded patients who underwent reduction mammoplasty for oncologic purposes as well as those who were deceased, did not have functional telephone numbers, or we were unable to contact them for the survey.

Institutional review board (IRB) approval was granted prior to the initiation of the study. In compliance with the guidelines outlined in the IRB approval, letters were mailed to potential participants to inform them of the study and give them the option to opt out. Eligible patients were surveyed using a customized questionnaire to assess long-term outcomes after reduction mammoplasty. The questionnaire (Table 1) was designed to capture patient perception of the most common symptoms reported by patients seeking reduction mammoplasty as well as aesthetic satisfaction and overall satisfaction following the procedure. The survey was designed to be brief so that it could be administered over the phone without undue inconvenience to study subjects. The survey was then administered over the phone to patients who did not opt out. Study data were entered into an electronic database. Postoperative outcomes including symptom relief, residual symptoms, change in breast size, nipple sensation, additional breast surgeries, abnormal postoperative mammography, weight gain, exercise tolerance, and ability to breastfeed were assessed. Analysis of variance was used to test for group differences in aesthetic and overall satisfaction. A value of *P* < .05 was considered statistically significant. All other categorical outcomes of interest were compared using χ^2^ analysis.

## RESULTS

### Preoperative and perioperative analyses

In total, 176 eligible patients were contacted by the phone for participation. Fifty of these (29.5%) patients declined to participate in the survey; 124 patients (70.5%) who were contacted completed the survey. All completed surveys were included in the statistical analysis. The mean age of patients at the time of surgery was 38.5 years (SD = 13.7 years) and ranged from 17 to 71 years of age. All patients reported physical symptoms prior to surgery, which included back pain, bra strap shoulder grooving, intertrigo, posture problems, and breathing difficulties (Table 2). The most common symptoms were back pain and shoulder grooving.

### Long-term postoperative outcomes

Postoperative outcomes were assessed and are displayed in Table 3. Roughly one-third (31%) of all patients reported having abnormalities on mammography. However, only 3 patients reported malignant diagnoses from subsequent workup. All 3 patients subsequently underwent appropriate breast cancer treatment including mastectomy. Only 20 patients (16%) had children after their reduction mammoplasty, 6 (30%) of whom reported that they were able to breastfeed.

The patients were then divided into subgroups according to their length of follow-up: 5, 10 and 15 years postprocedure. The majority of patients in all 3 groups reported that macromastia symptoms were completely or mostly resolved after surgery (75% vs 82% vs 82%, *P* = .840). In the 5-year group, 19% of people reported residual symptoms compared with 36% of people in both the 10- and 15-year groups, although this did not reach significance (*P* = .100). A larger proportion of patients 10 (45%) and 15 (43%) years out from surgery reported return of symptoms compared with those 5 (19%) years out from surgery; however, this difference was not statistically significant (*P* = .36). There was a significant difference in the proportions of patients in all 3 groups who reported an increase in breast size since surgery (40% vs 70% vs 51%, *P* = .0297). Similarly, there was a difference in proportions of patients who reported an increase in body weight between all 3 groups (13% vs 42% vs 41%, *P* = .0017). Interestingly, patients in all 3 groups reported improved exercise tolerance after surgery but no group benefitted more than the others (63% vs 55% vs 74%, *P* = .215).

There was a significant difference in the proportion of patients who reported decreases in nipple sensation: 31%, 36%, and 54% of people reported decreased nipple sensation in the 5-, 10-, and 15-year groups, respectively (*P* = .0416). Only 8 people in the study (4.5%) had additional breast surgeries after their reduction mammoplasty. Only 2 patients in the study (1.6%) reported that they had undergone a subsequent reduction mammoplasty after the initial procedure. Both patients attributed recurrent macromastia symptoms to weight gain after the first surgery.

### Satisfaction

Multiple questions in the survey also addressed patient satisfaction after reduction mammoplasty (Table 4). In all 3 groups, more than 90% of participants reported that they were happy with the reduction mammoplasty (93% vs 91% vs 91%, *P* = .914). In the 5-year group, 90% had no regrets with the surgery compared with 79% in the 10-year group and 77% in the 15-year group, but this was not significant (*P* = .181). When asked whether they would want another reduction mammoplasty, only 17% to 24% of participants indicated that they would want another reduction, with no significant differences among the 3 groups (*P* = .689).

To further quantify satisfaction, participants were asked to rank their overall and aesthetic satisfaction after surgery using a 5-point Likert scale with 2 verbal anchors (1 was the least satisfied and 5 most satisfied). In the 5-year group, the median overall satisfaction was 4.5; in the 10-year and more than 15-year groups, overall satisfaction was 4 and 4, respectively. Median values for aesthetic satisfaction were 4, 5, and 4 for the 5-, 10-, and 15-year group, respectively. Overall, patient satisfaction after reduction mammoplasty was high and did not significantly decrease over time.

## DISCUSSION

Reduction mammoplasty can improve quality of life for patients with macromastia by alleviating physical symptoms, increasing psychosocial well-being, and enhancing functional status.^4^^,^^6^^-^8 By utilizing patient-reported outcome measures to assess the efficacy of reduction mammoplasty, it is possible to evaluate the impact of surgery on patient satisfaction and quality of life with the goal of improving patient care. Using our customized questionnaire created specifically to assess outcomes after reduction mammoplasty, we demonstrate that women who undergo reduction mammoplasty experience symptom relief and exercise tolerance as well as high levels of satisfaction that is sustained for more than 15 years postoperatively. This is the first demonstration of longevity of patient satisfaction and symptom relief for more than 15 years following reduction mammoplasty.

Our patients reported high overall and aesthetic satisfaction across all groups. This study adds to the literature^3^ that demonstrates that reduction mammoplasty provides symptomatic relief and has additional benefits such as improvement in exercise tolerance. Our data showed that patients continue to report improvement in exercise tolerance after reduction mammoplasty for as long as 15 years. These changes may account for the improved quality of life after reduction mammoplasty described in previous studies.^7^^,^^13^^,^^15^^,^^16^ Reduction mammoplasty has also been demonstrated to have positive effects on sexual function, depression, self-esteem, and capacity to work.^17^^,^^18^

Interestingly, we also found that patients who were more removed from surgery reported a significant increase in breast size and body weight, which suggests that increases in breast size parallel weight gain. Weight gain is influenced by external factors such as changes in lifestyle, diet, and exercise, as well as hormonal changes associated with aging and pregnancy when applicable. Despite these changes in weight and increases in breast size, patients were still very satisfied with having breast reduction surgery. We did not survey for BMI to investigate the correlation between BMI and reduction mammoplasty satisfaction, as previous studies have demonstrated that these two variables are often independent.^10^ Future studies may investigate the trend of increasing weight gain with increased time removed from reduction mammoplasty, as this may influence patient satisfaction.

Our study provides evidence for the longevity of the benefits of reduction mammoplasty. It shows that patients’ high satisfaction is maintained for more than 15 years after the original procedure and appears to have a positive impact on patients’ overall quality of life. To our knowledge, this is the first demonstration of sustained patient satisfaction and symptom relief for more than 10 years following reduction mammoplasty.

### Limitations

Although this retrospective study provides insight into the long-term effects of reduction mammoplasty, it does have limitations that include recall bias and use of a retrospective, nonvalidated survey. Errors in recall may be more prevalent as time has elapsed since surgery. Furthermore, our study only assesses postoperative patient satisfaction. Prior studies compare preoperative survey results with postoperative results.^5^^,^^10^^,^^11^^,^^15^ Because of the retrospective nature of this review, we did not have the opportunity to survey patients prior to their surgeries. Thus, all results are based on the patients’ recollection of their quality of life and symptoms before and after surgery. Nevertheless, patients’ perceptions remain one of the most important measures of surgical outcomes.

Several studies investigating patient-reported outcomes after reduction mammoplasty have been conducted using the BREAST-Q.^11^ We chose to implement a novel, focused survey with questions specifically tailored to evaluate long-term symptom relief and satisfaction following reduction mammoplasty. The survey was designed to be brief so that it could be effectively administered over the phone to maximize completion rate and targeted responses. Although our questionnaire has not been formally validated, it provided a focused and practical instrument to evaluate patient satisfaction following reduction mammoplasty for the purpose of this study. In addition to symptom relief and satisfaction that are addressed on instruments such as the BREAST-Q, it evaluates patient-reported functional outcomes such as change in exercise tolerance. With these limitations in mind, this study adds to the literature as a concise custom study to allow for greater participation, collection of more specific data, and ease of execution, while showing the longevity of patient satisfaction for more than 15 years.

We also acknowledge the possibility of selection bias, as records of those with more historic surgeries are stored on paper charts or older electronic chart systems and are thus less easily retrieved. We did not have the ability to collect data of BMI, surgical technique, and postoperative complications because many of the surgeries occurred more than 10 years ago and these paper records have either not captured these data or records could not be accessed. Future studies could investigate the relationship of these factors with patient satisfaction.

## CONCLUSIONS

Reduction mammoplasty has long-lasting benefits for patients with macromastia and provides increased quality of life. Overwhelmingly, patients report satisfaction with the procedure and marked symptom relief that is sustained for as long as 15 years after surgery.

## Figures and Tables

**Table 1:** Survey questions on long-term outcomes following reduction mammoplasty

*Pre/perioperative outcomes*				
What symptoms did you have before the breast reduction?				
Rash	Posture issues			
Back pain	Breathing problems			
Shoulder grooving	Other			
*Postoperative outcomes*				
Did the breast reduction alleviate all or most of your symptoms?				
Completely	Mostly	Moderately	No change	Worse
Which of these symptoms were improved?				
Rash	Posture issues			
Back pain	Breathing problems			
Shoulder grooving	Other			
Have any of these symptoms returned?				
Rash	Posture issues			
Back pain	Breathing problems			
Shoulder grooving	Other			
What were the benefits of the surgery? (open ended)				
Have your breasts gotten larger since your reduction? Yes or No				
Were you able to breastfeed after the breast reduction, if applicable? Yes or No				
How has your nipple sensation changed?				
Increased	Decreased	No change		
Have you had any other breast surgery since your breast reduction? Yes or No				
Have you had any abnormalities on mammography after your breast reduction? Yes or No				
How has your weight changed since the reduction?				
Increased	Decreased	No change		
How has your exercise tolerance changed since the reduction?				
Increased	Decreased	No change		
Satisfaction				
Are you happy that you had your breast reduction? Yes or No				
Do you have any regrets with the surgery? Yes or No				
Do you feel as though you need another breast reduction? Yes or No				
On a scale of 1-5, how satisfied are you with the result? (1—Least satisfied; 5—Most satisfied)				
How pleased are you with the aesthetic result on a scale of 1-5? (1—Least satisfied; 5—Most satisfied)				

**Table 2:** Physical symptoms before surgery

Symptoms before surgery	5 y (n = 52)	10 y (n = 33)	15 y (n = 39)
Back pain	49 (94%)	29 (88%)	33 (80%)
Shoulder grooving	39 (75%)	29 (88%)	34 (90%)
Rashes	25 (48%)	6 (18%)	15 (38%)
Posture problems	29 (56%)	13 (39%)	8 (21%)
Breathing difficulty	11 (21%)	3 (9%)	4 (10%)

**Table 3:** Patient-reported outcomes after reduction mammoplasty

Postoperative outcomes	5 y (n = 52)	10 y (n = 33)	15 y (n = 39)	*P*^a^
Symptom relief	39 (75%)	27 (82%)	32 (82%)	.840
Residual symptoms	10 (19%)	12 (36%)	14 (36%)	.100
Breasts have enlarged	21 (40%)	13 (70%)	20 (51%)	**.0297**
Decreased nipple sensation	16 (31%)	12 (36%)	21 (54%)	**.0416**
Additional breast surgery	1 (2%)	4 (12%)	3 (8%)	.105
Abnormalities on mammogram	17 (33%)	10 (30%)	12 (31%)	.952
Benign	15 (88%)	10 (100%)	11 (92%)	.549
Weight gain	7 (13%)	14 (42%)	16 (41%)	**.0017**
Improved exercise tolerance	33 (63%)	18 (55%)	29 (74%)	.215

^a^ Bold values denote statistical significance.

**Table 4:** Patient satisfaction after reduction mammoplasty

Satisfaction	5 y (n = 52)	10 y (n = 33)	15 y (n = 39)	*P*
Happy with surgery	53 (93%)	30 (91%)	35 (91%)	.914
No regrets	47 (90%)	26 (79%)	30 (77%)	.181
Desired another reduction	9 (17%)	8 (24%)	9 (23%)	.689
Overall satisfaction, median	4.5	4	4	.226
Aesthetic satisfaction, median	4	5	4	.117
